# Upon entering an age of global ivermectin-based integrated mass drug administration for neglected tropical diseases and malaria

**DOI:** 10.1186/s12936-017-1830-z

**Published:** 2017-04-24

**Authors:** Frank O. Richards

**Affiliations:** River Blindness, Lymphatic Filariasis and Schistosomiasis Programs, The Carter Center, 453 Freedom Parkway, Atlanta, GA 30307 USA

## Abstract

Ivermectin mass drug administration (MDA) in humans to reduce malaria vectors is yet another use for this remarkable medicine whose discoverers shared the 2015 Nobel Prize in Medicine with the discoverer of artemisinin. The malaria community should join those who have long used ivermectin MDA in an integrated battle to break transmission of three vector-borne parasitic diseases.

This Thematic series of the *Malaria Journal* provides in depth and exciting information about a developing body of knowledge for the novel use of ivermectin mass drug administration (MDA) to reduce *Anopheles* vector numbers and block transmission of malaria. This is yet another example of the broad utility of a remarkable medicine, the discoverers of which co-shared the 2015 Nobel Prize in Medicine with the discoverer of artemisinin [1]. It is fitting that the malaria community reflect on the opportunity to engage with those who have conducted a two decade long ivermectin MDA campaign against onchocerciasis and lymphatic filariasis (LF) [2–4].

The onchocerciasis and LF communities have found a common branding (‘Neglected Tropical Diseases’-‘NTDs’) and strategy (ivermectin-based ‘Preventive Chemotherapy’) [5]. Donor funding for the greater NTD Preventive Chemotherapy initiative has been leveraged by Big Pharma drug donation programmes that have provided billions of safe and effective tablets, including ivermectin (Mectizan^R^, Merck & Co., Inc.) for onchocerciasis and LF, albendazole (GlaxoSmithKline) for LF and soil transmitted helminthiases (STH), diethylcarbamazine (Eisai) for LF, mebendazole (Johnson & Johnson) for STH, azithromycin (Zythromax^R^, Pfizer) for trachoma, and praziquantel (Merck KGaA) for schistosomiasis [5, 6].

The NTD approach to MDA can be roughly grouped into two camps: (1) Those whose STH and schistosomiasis MDA targets children (similar to seasonal malaria prophylaxis) primarily through school-health programmes, with most resources flowing through ministries of education, and; (2) Those whose MDA programmes aim to treat both children and adults for onchocerciasis, LF, and/or trachoma though community-wide strategies executed by ministries of health, where distributors provide treatment annually or semi-annually from a central post or by moving house to house [5]. It is this second strategy that should interest those in the malaria community who wish to deploy ivermectin. Hundreds of millions of doses of ivermectin have been provided in malarious areas through these NTD logistic systems for onchocerciasis since 1988 [2, 7], and (on the African continent combined with albendazole) for LF since 2000 [3]. As a result, at least two generations in the targeted populations are used to being offered free ivermectin tablets by health officials, or by volunteer distributors who are often friends and neighbours. The noticeable impact of these treatments on scabies, lice and intestinal worms makes ivermectin distribution a desirable and popular service. “Bring us our ivermectin” is the usual message [8].

The history of the use of ivermectin in the battle against onchocerciasis and LF is one of integration. Ivermectin-based MDA for onchocerciasis was integrated with LF (by adding albendazole) when that initiative was launched in Africa [9]. In many areas in Africa these two vector-borne filarial infections are co-endemic so that the ivermectin component of the MDA simultaneously treats both conditions; the proverbial killing two birds with one stone [10]. Use of LF drug distributors to also provide long-lasting insecticidal nets (LLIN) made strategic sense since the same LF *Anopheles* vectors transmit malaria [11]. Fighting anaemia by simultaneously targeting both malaria and hookworm (with LLIN, ivermectin and albendazole) was another “two-fer” [12, 13].

It is time to consider expanding ivermectin MDA through an NTD-malaria ivermectin alliance. The onchocerciasis, LF and malaria communities agree that transmission interruption can only be achieved by maximizing treatment coverage. They have a common interest in strengthening community-based logistical systems so that ivermectin can flow with increasing frequency and efficiency to villages at the end of the road. The expanded frequency and scope of ivermectin use (or the deployment of a long-lasting ivermectin preparation) proposed by the malaria community will surely lead to a quicker end for onchocerciasis and LF in Africa. Outside of Africa, the LF programme hopes to exploit ivermectin’s recently discovered synergy with diethylcarbamazine and albendazole to provide an apparent single dose LF ‘cure’ [14]. An expansion of ivermectin use in Asia, the Pacific and the Americas could likewise result in an earlier end to LF and malaria there.

There are a number of challenges to a vision of global integrated ivermectin-based MDA, only two of which I will mention in this commentary. The first is the need for further research on how to minimize the rare but serious adverse events (stupor and coma) that can result from first ivermectin treatment in individuals infected with the filarial parasite *Loa loa* in forested areas of central Africa [15, 16]. These SAEs have restricted ivermectin use in some of the most malarious areas on the planet. The second is the challenge of enhancing the global supply of ivermectin. Beyond projected increases in consumption by onchocerciasis, LF and malaria programmes, there are new calls for community-wide ivermectin MDA for scabies [17] and strongyloidiasis [18]. It is unreasonable if not unfair to expect that the emerging global demand for ivermectin can be fully met through the generosity of Merck & Co. and its Mectizan Donation Program [19]. Funding to manufacture this ‘miracle medicine’ will likely have to be found elsewhere if we are bring forth a vision of pluripotent and global ivermectin mass drug administration.
